# Infection of bovine well-differentiated airway epithelial cells by *Pasteurella multocida*: actions and counteractions in the bacteria–host interactions

**DOI:** 10.1186/s13567-020-00861-2

**Published:** 2020-11-23

**Authors:** Ang Su, Jie Tong, Yuguang Fu, Sandy Müller, Yenehiwot Berhanu Weldearegay, Paul Becher, Peter Valentin-Weigand, Jochen Meens, Georg Herrler

**Affiliations:** 1 Institute of Virology, University of Veterinary Medicine Hannover, Foundation, 30559 Hannover, Germany; 2 Institute of Microbiology, University of Veterinary Medicine Hannover, Foundation, 30559 Hannover, Germany; 3 State Key Laboratory of Veterinary Etiological Biology, Lanzhou Veterinary Research Institute, Chinese Academy of Agricultural Sciences, Lanzhou, 730046 China; 4 College of Life Science, Institute of Life Science and Green Development, Hebei University, Baoding, 071002 China

**Keywords:** *Pasteurella multocida*, air–liquid interface (ALI) cultures, bacterial pathogenesis, host–pathogen interactions

## Abstract

*Pasteurella (P.) multocida* is a zoonotic pathogen, which is able to cause respiratory disorder in different hosts. In cattle, *P. multocida* is an important microorganism involved in the bovine respiratory disease complex (BRDC) with a huge economic impact. We applied air–liquid interface (ALI) cultures of well-differentiated bovine airway epithelial cells to analyze the interaction of *P. multocida* with its host target cells. The bacterial pathogen grew readily on the ALI cultures. Infection resulted in a substantial loss of ciliated cells. Nevertheless, the epithelial cell layer maintained its barrier function as indicated by the transepithelial electrical resistance and the inability of dextran to get from the apical to the basolateral compartment via the paracellular route. Analysis by confocal immunofluorescence microscopy confirmed the intactness of the epithelial cell layer though it was not as thick as the uninfected control cells. Finally, we chose the bacterial neuraminidase to show that our infection model is a sustainable tool to analyze virulence factors of *P. multocida*. Furthermore, we provide an explanation, why this microorganism usually is a commensal and becomes pathogenic only in combination with other factors such as co-infecting microorganisms.

## Introduction

Bovine respiratory disease is a multifactorial disease complex of cattle (BRDC) [1–3]. *Pasteurella multocida* is one of the most important bacterial pathogens related to BRDC [3–5]. With its broad host range comprising humans, domestic animals and wild animals [1, 6, 7], *P. multocida* is considered as one of the most prevalent commensals and opportunistic pathogens worldwide [7, 8]. As a commensal of cattle, *P. multocida* is located in the upper respiratory tract [1, 9–11]; the pathogenesis of the respiratory disease and the interactions with other respiratory pathogens are largely unknown. Several bacterial components have been identified as virulence factors, e.g. the capsule, lipopolysaccharides, and the neuraminidase (sialidase) [8, 12–17]. A neuraminidase is found in most *P. multocida* strains [14, 18]. By releasing sialic acid from glycosylated host proteins and lipids, the enzyme provides a carbon source for bacterial amplification [19, 20]; furthermore, it may help to escape the host defense mechanisms by releasing sialic acid from mucins [21], a crucial component of the ciliary clearance function [22, 23].

The airways are lined by a layer of epithelial cells that form a primary barrier to invading respiratory pathogens. Mucins released by mucus-producing cells can entrap detrimental substances including microorganisms [24, 25], which are then transported out of the respiratory tract by the coordinated movement of the cilia present on ciliated cells [24, 26]. Another crucial component of the airway epithelium is the basal cells, which ensure the regeneration capacity when part of the cells have been lost, e.g. by damage due to environmental material [27]. To maintain the barrier function, the epithelial cells have a polarized organization. A characteristic feature of the cellular polarity is that the plasma membrane is divided into an apical domain facing the environmental side of the epithelium and a basolateral domain facing the internal milieu. The two domains have a different composition and are separated by tight junctions that not only prevent an intermixing of the components of the two membrane domains but also form a tight connection to the neighboring cells, which prevent an invasion of pathogens via the paracellular route [24]. For many microorganisms, the role of the respiratory epithelium during infection remains largely unknown. As a close in vitro representation of the airway epithelium, air–liquid interface (ALI) cultures of well-differentiated epithelial cells have been utilized to analyze infection by different pathogens including viruses and bacteria [27–31].

We have applied filter-grown cultures of bovine well-differentiated airway epithelial cells to analyze the bacterial infection. *P. multocida* grew readily on the bronchial epithelial cells cultures and induced a substantial loss of ciliated cells. Despite this loss, the epithelial cell layer maintained the barrier function as indicated by the transepithelial electrical resistance (TEER). This was achieved by a reorganization process that resulted in a reduced thickness of the epithelial cell layer. Furthermore, we present evidence that the bovine ALI cultures can be used to analyze the role of the bacterial neuraminidase as a virulence factor.

## Materials and methods

### Differentiated bovine airway epithelial cell cultures

Fresh lungs were collected from calves slaughtered at a local slaughterhouse in Germany. Bovine primary bronchial epithelial cells (PBEC) were isolated as previously described [30] and were expanded in growth medium (BEGM). When the PBEC reached confluence, the cells were transferred to Transwell® polycarbonate membranes (Corning) and maintained under the air–liquid interface (ALI) conditions for at least 4 weeks at 37 °C in a humidified 5% CO_2_ atmosphere. During this time, the cells grew to a pseudostratified monolayer; as the nuclei were located at different heights, the cells appeared to have a multilayered organization. While all differentiated cells have contact to the filter substrate, there are several basal cells interspersed at the bottom of the monolayer [32].

### Bacterial strain and growth conditions

The applied bacterial strain in this study was *P. multocida* strain 1701. *P. multocida* 1701 is a field strain, isolated from purulent nasal exudate of dairy cattle [33]. The strain belongs to the capsular serogroup A, which is the most prominent type in bovine [34, 35]. For the profiling of virulence associated genes, we used a multiplex PCR assay [33, 36, 37], and two additional single gene PCR´s for *hgbB* and *tbpA *[34]. The results indicated, that the strain harbors most of the tested genes, including the two sialidase encoding genes *nanH* and *nanB*, but not the dermonecrotic toxin gene *toxA* (Table 1). Based on published methods [38], the recovered cryo-stocks bacteria were used for the infection experiment. Bacteria were recovered from cryo-stocks according to the following protocol. Cryo-conserved *P. multocida* were grown on Columbia agar supplemented with 7% sheep blood (Oxoid) overnight under aerobic conditions at 37 °C. One colony of *P. multocida* from a blood agar plate was inoculated into BHI broth and incubated overnight at 37 °C with shaking. Afterwards, 20 mL fresh BHI medium was inoculated with the overnight broth culture to an initial OD_600_ of 0.05. This was allowed to grow for 4–5 h until it reached an OD_600_ of approx. 2.5 ± 0.3, which was found to be the mid of the logarithmic growth phase. The number of bacteria was determined by plating serial tenfold dilutions on Columbia agar plates supplemented with 7% sheep blood. Bacterial titers are presented as CFU per milliliter (CFU/mL).

**Table 1:** **Virulence associated genes and capsule types detected in** ***P. multocida* strain 1701**

Process or enzyme	Gene	Gene test	Reference
Capsule biosynthesis (CapA)	*hyaD-hyaC*	+	[37]
Dermonecrotic toxin	*toxA*	−	[36]
Outer membrane protein	*omA87*	+	[36]
	*ompH*	−	[36]
	*ompA*	−	[36]
	*plpB*	+	[36]
Sialidases	*nanH*	+	[36]
	*nanB*	+	[36]
Adhesins	*ptfA*	+	[36]
	*pfhA*	+	[36]
Iron metabolism	*tonB*	+	[36]
	*hgbA*	+	[36]
	*hgbB*	−	[34]
	*tbpA*	+	[34]

In ‘Gene test’ column, ‘ + ’ represent to positive for the gene test and ‘ −’ to  negative.

### Bacterial infection of well-differentiated epithelial cells

Well-differentiated PBECs were kept in the absence of antibiotics and antimycotics 24 h prior to bacterial infection. The cell number per filter support was approximately 5 × 10^5^. Transwell filters were washed five times with warm PBS and infected with *P. multocida* inoculum in 100 µL final volume added to the apical surface at three different conditions: 10^2^ CFU for 1 h, 10^2^ CFU for 4 h, 10^5^ CFU for 4 h. Control cells were mock-infected with PBS. After the inoculation period, PBEC were rinsed with warm PBS twice to remove unbound bacteria and fresh ALI medium without antibiotics and antimycotics was added only at the basolateral compartment. Infected PBEC were incubated for up to 24 h under ALI conditions at 37 °C and 5% CO_2_. As there was no medium present in the apical compartment, bacteria could grow only on nutrients derived from the mucus-covered epithelial cell layer, similar to natural infections. At 4, 12, and 24 hpi, 100 μL of ALI medium were applied apically and the cultures were incubated for 30 min at 37 °C. After removal of the supernatant, cells were kept again under ALI conditions until the next time point of sample collection. Replicate plating of supernatants was performed with tenfold serial dilutions on Columbia agar supplemented with 7% sheep blood to determine the bacterial growth kinetics.

### Measurement of the barrier integrity

Respiratory epithelial cells were grown under ALI conditions. The transepithelial electrical resistance (TEER) was determined by using the Millicell® ERS-2 Voltohmmeter (Millipore) according to the manufacturer’s instructions.

To determine the permeability for macromolecules, FITC-labelled dextran (70 kDa) (Invitrogen) was applied to the apical compartment. Medium was collected from the basolateral compartment at different time points, and analysed for fluorescence with a spectrophotometer (Varian Cary Eclipse).

### Cytotoxicity assay

Filter-grown cultures of well-differentiated airway epithelial cells were used to determine the cytotoxic of effect of the bacterial infection. For the determination of the cytotoxicity effect, 70 μL PBS were added to the apical compartment and cells were incubated on a horizontal shaker for 5 min. Supernatants were collected at different time points and analyzed for the release of LDH by using the Cytotx® 96 assay kit (Promega). All the experiments were performed at least three times.

### Analysis of the importance of the neuraminidase

To analyze the importance of the neuraminidase of *P. multicida*, cells were either pretreated with exogenous neuraminidase or incubated in the presence of a neuraminidase inhibitor. Bovine epithelial cells were treated with neuraminidase from *C. perfringens* (Sigma-Aldrich) at 200 mU for 1 h at 37 °C prior to bacterial infection. After removal of the enzyme by washing three times, 100 CFU of bacteria in 100 μL medium were applied. After 1 h, the infection was continued as described above. For the inhibition experiment, the neuraminidase inhibitor (NAI) DANA (Sigma-Aldrich) was added at a final concentration of 1 mM to the inoculum and to the maintenance ALI medium up to 24 hpi. Supernatants were collected at 4, 12, 24 hpi to determine the cytotoxicity, the bacterial growth kinetics and for immunofluorescence microscopy.

### Immunofluorescence microscopy

All infected and mock-infected samples were washed with PBS three times and fixed with 3% paraformaldehyde (PFA) for 20 min. PFA was removed and 0.1 M glycine was added for 5 min. Samples were permeabilized with 0.2% Triton X-100, washed three times with PBS and were further blocked with 5% goat serum and incubated with a primary and a secondary antibodies consecutively for 1 h each. After washing with PBS, the nuclei were stained with DAPI (4′,6-diamidino-2-phenylindole) and embedded in Prolong Gold Antifade Reagent (Life Technologies), and stored at 4 °C for further analysis.

*P. multocida* infected samples and control samples were stained with Cy3-labeled antibody against β-tubulin (1:300, Sigma) as primary antibodies. Samples were analyzed by using an inverse microscope Nikon Eclipse Ti-S (Nikon) and TCS SP5 confocal laser scanning microscope (Leica). Images were analyzed using NIS-Elements Viewer 4.20 software (Nikon), while imaging analysis of cilia coverage rates to the well-differentiated cells was calculated by using ImageJ.

### Statistical analyses

All in vitro experiments were performed at least three times and data were analyzed using GraphPad Prism (GraphPad Software version 8, San Diego, CA, USA) software with Tukey multiple comparison test. Results were shown as means with standard deviations. A p-value of < 0.05 was considered significant.

## Results

### Growth kinetics of* P. multocida* on differentiated bovine airway epithelial cells

**Figure 1:**
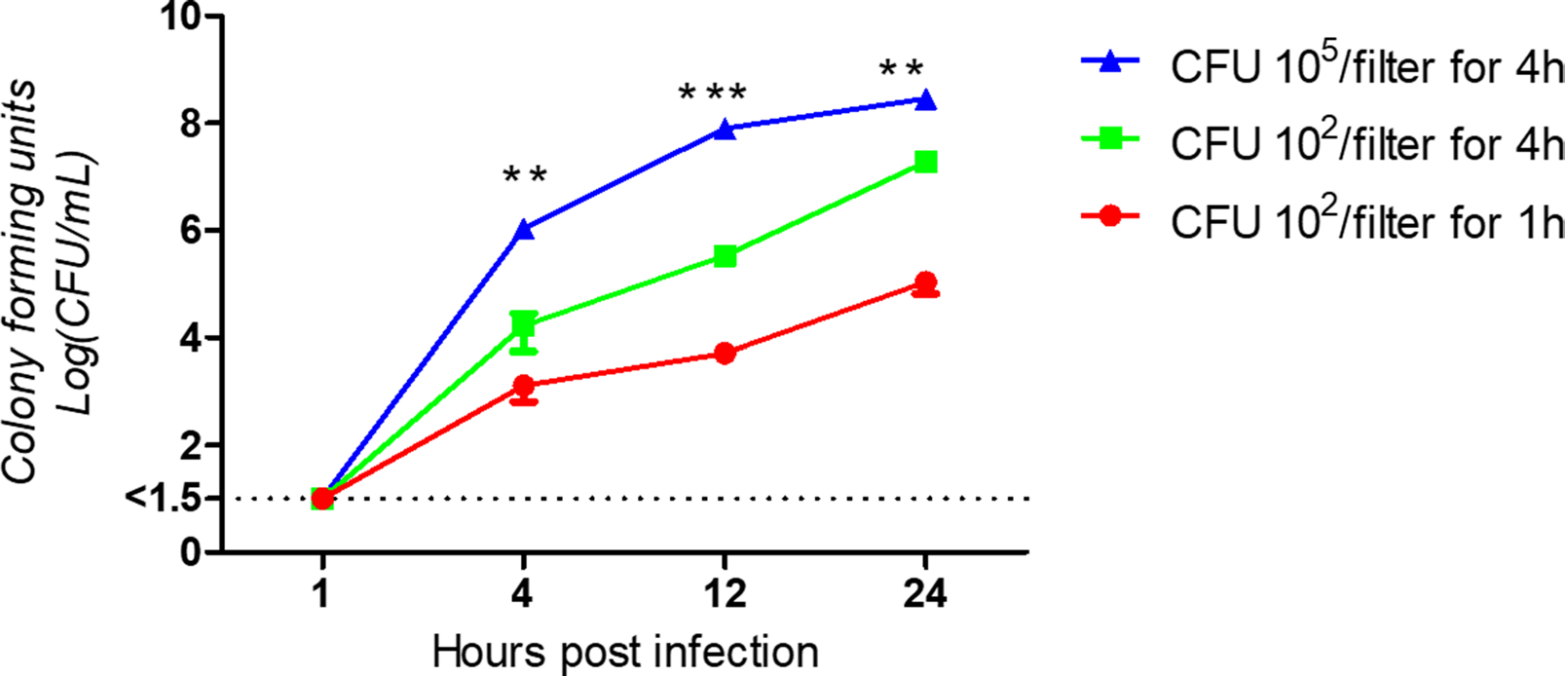
**Growth kinetics of** ***P. multocida* in well-differentiated bovine airway epithelial cell cultures.** Filter-grown well-differentiated bovine bronchial epithelial cells were infected with *P. multocida* by inoculation with 10^2^ or 10^5^ CFU for 1 h or 4 h. Supernatants were harvested at 4, 12 and 24hpi and used to determine the growth kinetics. The significance of the differences between the values in each group is indicated with asterisks by comparing the CFU titer vertically. Results represent the mean values of CFU ± SEM determined from three independent experiments with duplicated samples. ***P < 0.001, **P < 0.01 and*P < 0.05.

To determine the growth kinetics of *Pasterella multocida* on differentiated airway epithelial cells, filter-grown bovine ALI cultures were infected by strain 1701 (for virulence profile see Table 1 and Materials and Methods) from the apical side. Supernatants were collected at 4, 12 and 24 h post-infection and used to determine the colony forming units (CFU). Between the time points of sample collection, the cells were maintained under air–liquid interface conditions. As shown in Figure 1, *P. multocida* grew readily under the ALI culture conditions. Bacterial replication was dependent on the number of CFU applied in the inoculum and on the inoculation time. The sample with the most favorable conditions (10^5^ CFU, 4 h) grew to a concentration of 10^6^ CFU/mL by 4 h post infection (hpi); to reach the same concentration, it took more than 12 h for the second group (10^2^ CFU, 4 h) and more than 24 h for the third group (10^2^ CFU, 1 h) (Figure 1). It should be noted that in this experimental setup, bacteria were kept in the absence of media in the apical compartment. Therefore, bacterial growth was dependent on nutrients derived from the mucus-covered epithelial cell layer.

### Detrimental effect induced by* P. multocida* infection

**Figure 2:**
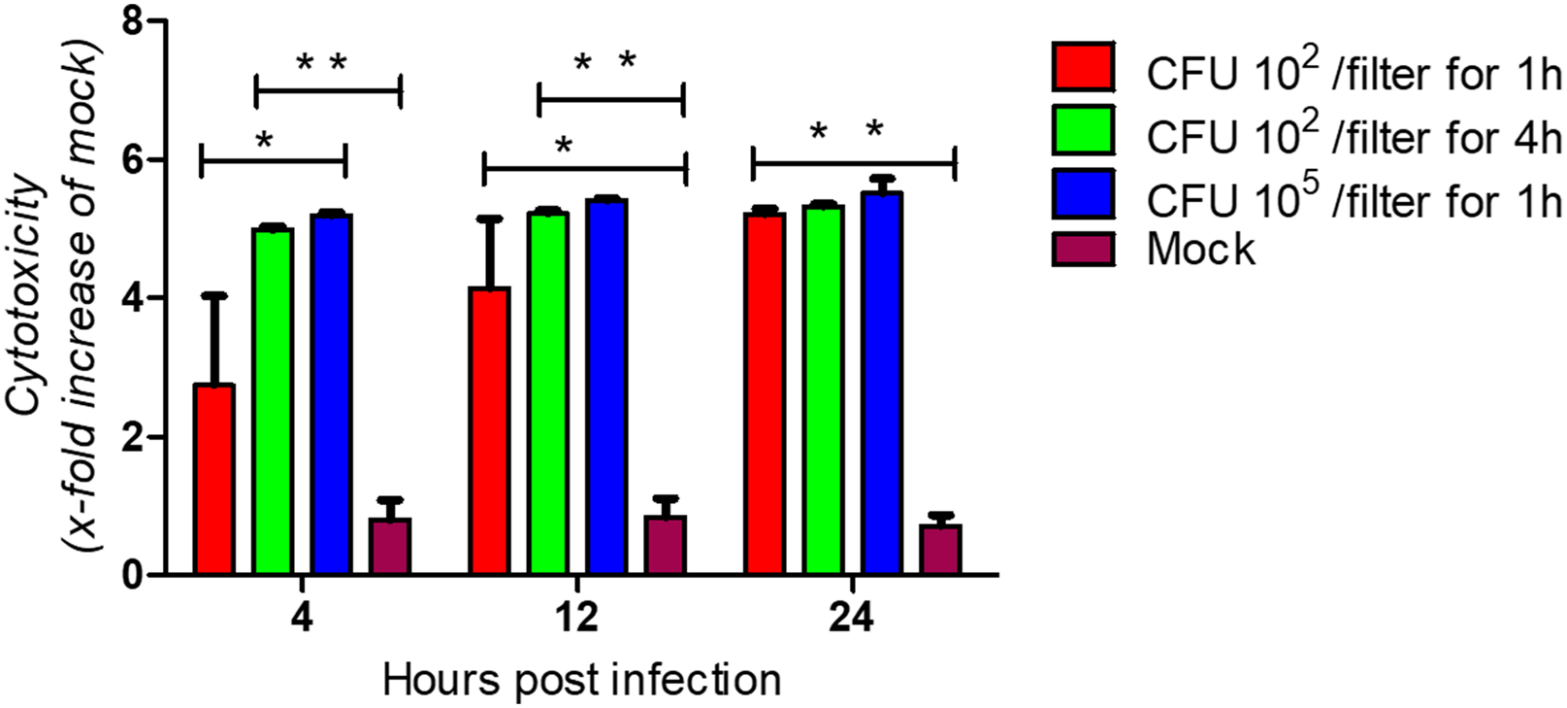
**Cytotoxic effect of** ***P. multocida* grown on well-differentiated bovine bronchial epithelial cells**. Filter-grown ALI cultures were infected by *P. multocida* at the indicated inoculation conditions (10^2^ or 10^5^ CFU, 1 h or 4 h). At the times indicated, the amount of LDH released into the apical compartment was determined.

Having shown that *P. multocida* is able to grow on differentiated airway epithelial cells, it was interesting to learn whether the bacterial infection has a detrimental effect on the respiratory epithelial cells. For this purpose, we analyzed the cytotoxic effect of *P*. *multocida*. Differentiated bovine airway epithelial cells were infected by 100 μL of strain 1701 from the apical side using the same inoculation conditions as described above for the growth curve (10^5^ CFU, 4 h; 10^2^ CFU, 4 h; 10^2^ CFU, 1 h). To get information about the cytotoxicity, we determined the amount of lactate dehydrogenase (LDH) released into the supernatant. As shown in Figure 2, in the supernatants of cells infected by *P. multocida* an increased amount of LDH was detected compared to mock-infected controls. The cytotoxic effect of the bacteria was time- and concentration-dependent.

**Figure 3:**
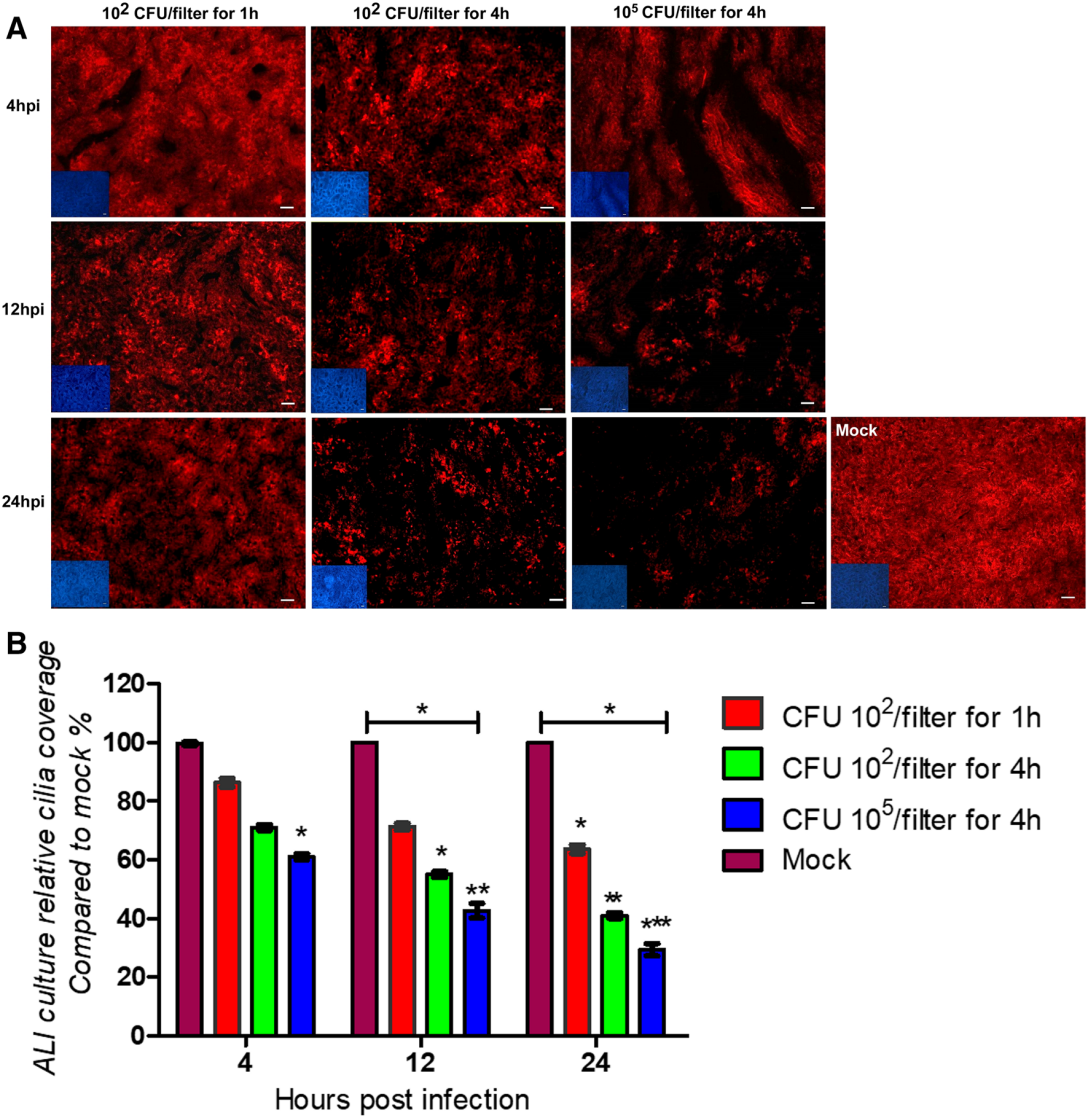
**Effect of** ***P. multocida* infection on ciliated cells.** Well-differentiated bovine airway epithelial cells were infected by *P. multocida* at the inoculation conditions indicated (10^2^ or 10^5^ CFU, 1 or 4 h). At 4, 12, 24hpi, samples were immune-stained (anti-β-tubulin, DAPI) to visualize cilia (red) and nuclei (blue). **A** Pictures obtained by microscopy; **B** Quantitation of the relative red fluorescence. Results are presented as mean ± SEM compared to mock-infected samples; Scale bars-50 µm.

To find out which cells were affected by the cytotoxic effect of *P. multocida*, we performed an analysis by confocal immunofluorescence microscopy. Bovine well-differentiated epithelial cells were infected by *P. multocida* 1701 strain. The filters were fixed at 4, 12, and 24 hpi and subjected to immunofluorescence staining. As shown in Figure 3A, the tubulin positive area of infected samples was reduced. The decreased level of red fluorescence was quantified and found to be significant (Figure 3B). This finding indicated that *P. multocida* infection resulted in the loss of ciliated cells. The decrease in the cilia staining was dependent on the number of bacteria applied and the inoculation time. In the sample with the highest bacterial load and longer initial infection time, only 30% of the cilia were visible 24 hpi and this difference is statistically significant compared with uninfected controls (p-value < 0.01) as well as the samples infected for 1 h with 100 CFU/filter (p-value < 0.05).

### Maintenance of the barrier function

**Figure 4:**
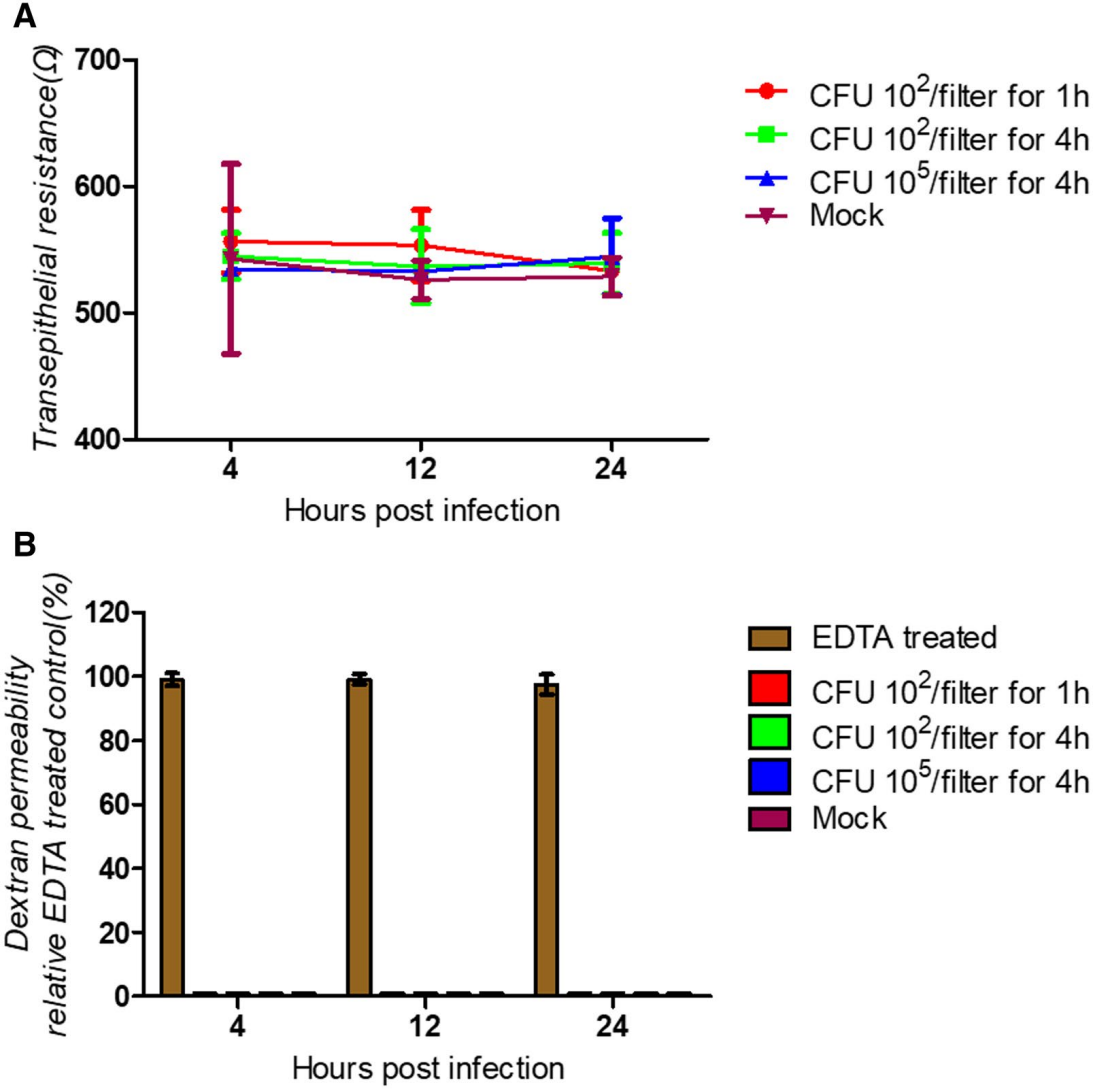
**Barrier function of bovine well-differentiated airway epithelial cells after infection by *P. multocida.*** Filter-grown ALI cultures of differentiated bovine airway epithelial cells were infected by *P. multocida* at the indicated inoculation conditions (10^2^ or 10^5^ CFU; 1 or 4 h). At the times indicated, samples were analyzed for the intactness of the barrier function **A** by determining the transepithelial electrical resistance (TEER) and **B** by determining the permeability for FITC-labeled dextran.

Next, we analyzed whether the loss of ciliated cells affects the barrier function of the epithelial cell layer. For this purpose, the transepithelial electrical resistance (TEER) was determined. As shown in Figure 4A, over a period of 24 hpi, none of the three inoculation scenarios resulted in a significant decrease. To confirm this result, a second assay was applied, which was based on the paracellular diffusion of dextran. As shown in Figure 4B, the macromolecule applied to the apical compartment was unable to reach the basolateral compartment, irrespective of the presence or absence of bacteria. Cells infected by *P. multocida* were found to have the same electrical resistance as mock-infected cells. By contrast, when the tight junctions were opened by treatment with EDTA, dextran was readily detected on the basolateral side of the cells.

**Figure 5:**
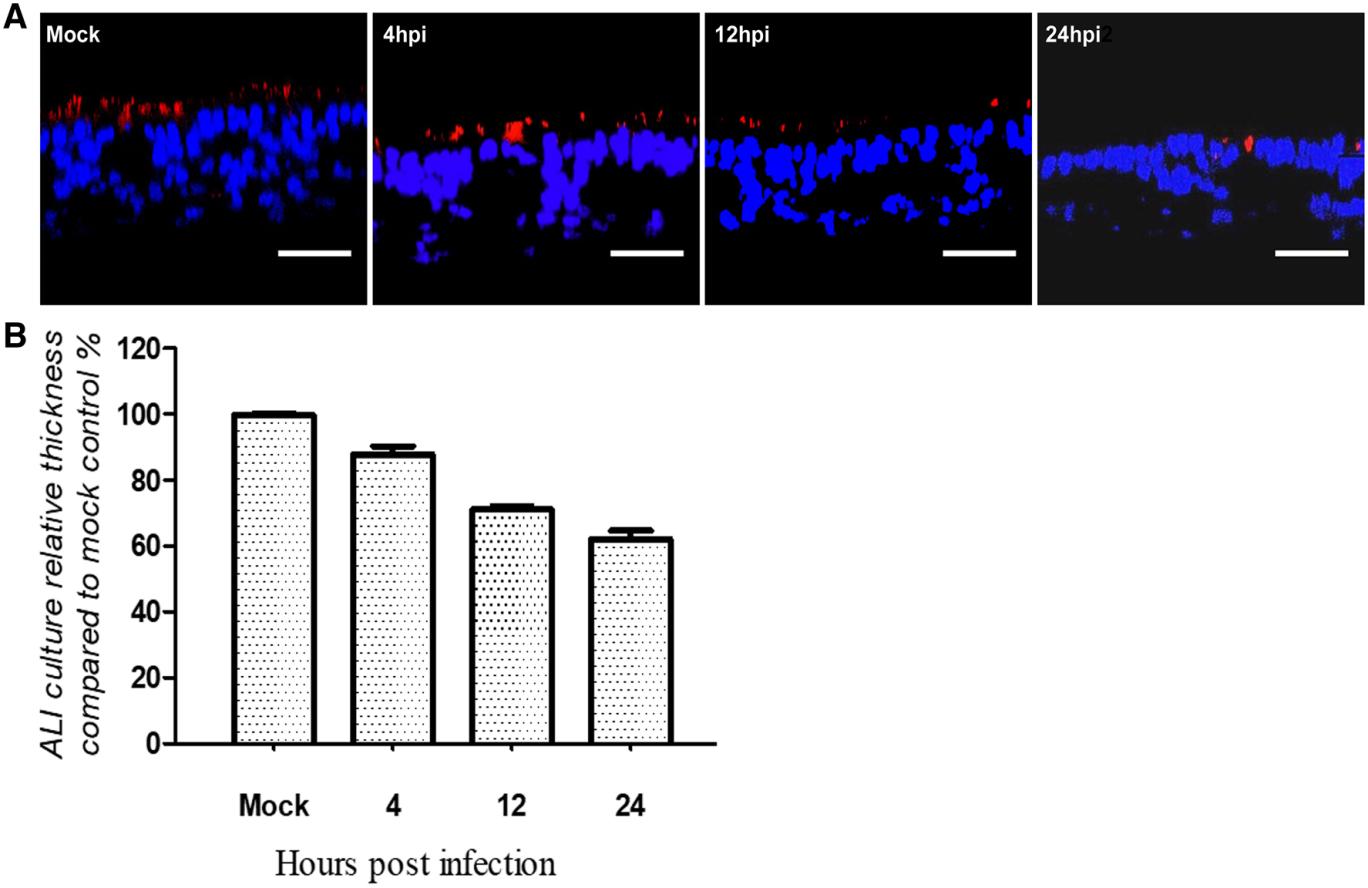
**Effect of infection by** ***P. multocida* on the thickness of the epithelial cell layer.** Differentiated bovine airway epithelial cells were infected by *P. multocida* at the indicated inoculation conditions (10^2^ or 10^5^ CFU; 1 or 4 h) and immune-stained as described in Figure 3. **A** Vertical sections of the immunostained samples; **B** relative thickness of the epithelial cell layer compared to mock infected samples. Scale bars, 50 µm.

To understand why the epithelial cells were able to maintain the barrier function despite a dramatic loss of ciliated cells, we analyzed vertical sections of confocal fluorescence micrographs. In Figure 5A, the staining of nuclei by DAPI, shows the picture that is characteristic for an intact epithelial layer. However, clear differences were observed between infected and mock-infected samples, with the most striking differences at 24 hpi, where very few ciliated cells were visible. The other difference observed was the considerable reduction in cell layer thickness, which was statistically significant and dependent on the length of the infection period. A reduction up to 30% was observed in infected samples after 24 hpi. This result suggests that the epithelial cells react upon the loss of ciliated cells by a rearrangement that results in a thinner cell layer but allows to maintain the barrier function.

### Importance of the bacterial neuraminidase for infection

**Figure 6:**
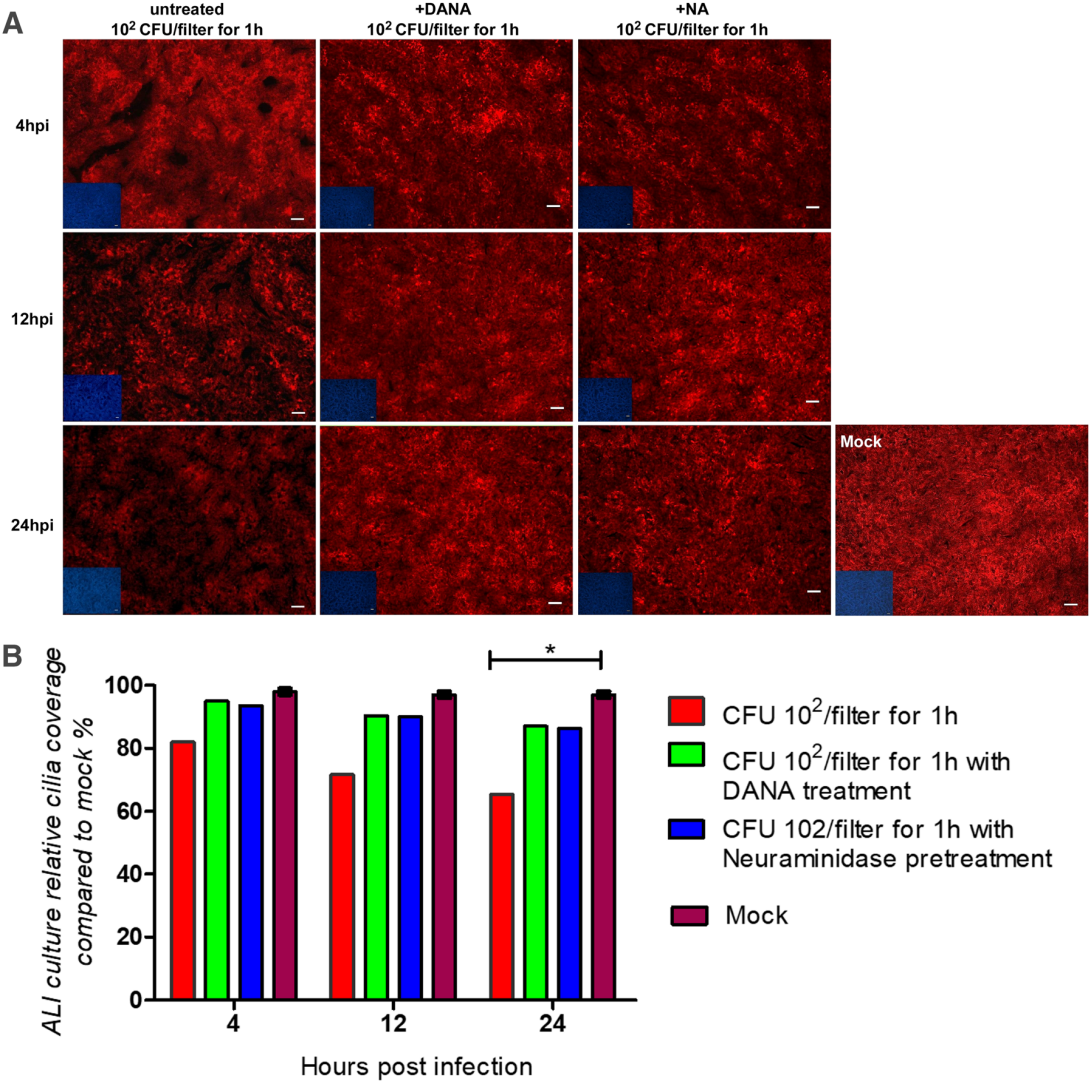
**Importance of the bacterial neuraminidase for** ***P. multocida* infection.** Filter-grown ALI cultures were infected by *P. multocida* (inoculation with 10^2^ CFU for 1 h). The importance of the bacterial neuraminidase was analyzed by pretreating some samples with neuraminidase from *C. perfringens* and by incubating some of the other samples in the presence of the neuraminidase inhibitor DANA. At 4, 12, 24 hpi, samples were immune-stained (anti-β-tubulin, DAPI) to visualize cilia (red) and nuclei (blue). **A** Pictures obtained by microscopy; **B** Quantitation of the relative red fluorescence. Results are presented as mean ± SEM compared to mock-infected samples.

Finally, we were interested to know whether the ALI-based infection model for *P. multocida* can be used to analyze potential virulence factors. For this purpose, we chose the neuraminidase of *P. multocida,* an enzyme, which is reported to support bacterial growth by releasing sialic acid from the apical surface of the airway epithelial cells to be used as a carbon source for the bacterial metabolism. To prevent this action, we applied two inhibition conditions: on one hand, addition of 2-deoxy-2,3-didehydro-N-acetylneuraminic acid (DANA, a neuraminidase inhibitor reagent) [39, 40] to directly prevent the enzyme activity and on the other hand, an indirect way to set the enzyme out of action by pretreating the cells with exogenous neuraminidase. In this way, sialic acids were released from the cell surface prior to infection and could not be used for bacterial growth. Therefore, the infection protocol was extended by including samples that either included the inhibitor DANA during the whole infection period or had been pretreated with neuraminidase from *C. perfringens*. For this purpose, we chose inoculation condition of 10^2^ CFU, 1 h. As reported above, infection of bovine ALI cultures by *P. multocida*, resulted in a loss of ciliated cells 24 hpi (Figure 6A). The cilia staining at this time point was reduced by more than 30% (red columns) and the difference was statistically significant (Figure 6B). Both the treatment with DANA (green columns) and the pretreatment with neuraminidase (blue columns) prevented this effect. Their values were only slightly lower compared to the mock-infected samples (brown columns) and these differences were not statistically significant. This result illustrates that the neuraminidase of *P. multocida* contributes to the detrimental effect of this pathogen in ALI cultures. Therefore, this infection model is an interesting tool to analyze virulence factors of this pathogen.

## Discussion

Though *P. multocida* is considered as one of the most prevalent commensals and opportunistic pathogens worldwide [7, 8], little information is available about the bacteria–host interactions. We applied an ALI culture system of bovine airway epithelial cells to analyze the interactions of *P. multocida* with differentiated respiratory epithelial cells. We were able to show that the bacteria were capable to grow very efficiently under ALI conditions. The efficient microbial replication resulted in a toxic effect, which was evident not only by the release of LDH, but also in a substantial reduction of the number of ciliated cells and cell layers. The loss of ciliated cells may be explained by bacteria-induced apoptosis [31]. This assumption is based on findings with ALI cultures infected by influenza viruses and *Streptococcus suis* [27, 31]. This infection model is also characterized by a loss of ciliated cells and it has been shown that the infected ciliated cells undergo apoptosis. Another similarity of the viral and the bacterial infection model is that the loss of ciliated cells is not associated with a loss of the barrier function of the epithelial cell layer [27, 31, 41]. Neither ions nor macromolecules can pass via the paracellular route from the apical to the basolateral side of the cells. A substantial reorganization of the remaining cells is required to maintain the TEER and the impermeability for DEAE-dextran. One possibility to compensate the loss of ciliated cells is that basal cells start to multiply and fill the gap. The full differentiation process, i.e. the acquisition of cilia may take more than two weeks. We hypothesize that the cells may adopt a polarized organization including tight junctions already early in the differentiation process and thus contribute to the barrier function. An alternative possibility is that only part of the ciliated cells are lost and others are undergoing a dedifferentiation process and thus contribute to the reorganization process required for maintaining the barrier function. Whatever possibility is true, our results show that not only the bacterial infection is efficient, but also the response of the epithelial cells is also efficient. The factors involved in the regeneration of the basal epithelial cells for maintaining barrier integrity remain to be further investigated.

Though the infected epithelial cell layer may maintain the barrier function, it takes some time until the compensation of ciliated cells is completed. In the transition phase, the area of infection lacks ciliated cells for some time and thus is not fully protected by the mucociliary clearance system. Therefore, these cells may be more susceptible to infection by other microorganisms; this assumption provides an explanation how *P. multocida* can facilitate co-infection and thus contribute to BRDC.

Under the conditions chosen for this study, the epithelial cells maintained the barrier function up to 24 hpi. When the bacteria were allowed to amplify for a longer time, a number of cells were lost which resulted in the decline of the TEER values indicating that the epithelium could not maintain its barrier function (not shown). This observation may raise the question why *P. multocida* usually is not pathogenic per se, but rather a commensal that becomes pathogenic only under special conditions. One reason for this may be that the ALI culture system has mucus and ciliary activity, but in contrast to the airways, the mucus is not transported out of the system and thus not removed from the epithelial cells. The clearance system of the host may keep the bacterial load at a low level and thus prevent the bacteria from becoming pathogenic. This explanation is supported by our finding that bacteria could be efficiently collected from the surface of the mucus-covered epithelial cells by washing with PBS. This result indicates that the binding of *P. multocida* is not very tight. In fact, after thorough washing only few bacteria were detected on the cell surface by immunostaining (not shown).

Our results show that the bovine ALI infection model can be used to analyze virulence factors of *P. multocida*. By applying the inhibitor DANA, we confirmed that the bacterial neuraminidase is crucial for efficient amplification. The function of this enzyme is thought to release sialic acid from cell surface macromolecules to be used as an energy source for the metabolism of *P. multocida* [42]. This view is supported by our second inhibitor scenario. When the cells were pretreated with an exogenous neuraminidase, the bacteria encountered desialylated cells and the enzyme of *P. multocida* had no substrate to release sialic acid and thus was unable to provide a substrate for the bacterial metabolism. While this manuscript was prepared for submission, a report was published in which ALI cultures of bovine airway epithelial cells were used to analyze the infection by a related pathogen, *Mannheimia haemolytica*. These authors found that the barrier function was maintained though *M. haemolytica* preferentially affected non-ciliated cells. Different from our study, in those experiments, infection was initiated with a high bacterial load. Under these conditions, *M. haemolytica* serotype A1, but not A2, became invasive and crossed the epithelial barrier by transcytosis [43]. This finding underscores that ALI cultures are a valuable tool that can provide results that cannot be obtained with immortalized cells.

Taken together, we have established an in vitro infection model that enables to analyze the interaction of *P. multocida* with its target cells in the respiratory tract. In this way, we could characterize the detrimental effect of the bacteria and the response of the airway cells. The ALI infection model was also used to characterize the importance of the bacterial neuraminidase and should be helpful in the future also for analyzing other virulence factors of *P. multocida*.

## Data Availability

The dataset supporting the conclusions of this article is included within the article (and its additional file(s)).
